# Generation and timing of graded responses to morphogen gradients

**DOI:** 10.1242/dev.199991

**Published:** 2021-12-17

**Authors:** Shari Carmon, Felix Jonas, Naama Barkai, Eyal D. Schejter, Ben-Zion Shilo

**Affiliations:** Department of Molecular Genetics, Weizmann Institute of Science, Rehovot 7610001, Israel

**Keywords:** *Drosophila*, Embryonic Development, Gastrulation, Morphogen, Transcription

## Abstract

Morphogen gradients are known to subdivide a naive cell field into distinct zones of gene expression. Here, we examine whether morphogens can also induce a graded response within such domains. To this end, we explore the role of the Dorsal protein nuclear gradient along the dorsoventral axis in defining the graded pattern of actomyosin constriction that initiates gastrulation in early *Drosophila* embryos. Two complementary mechanisms for graded accumulation of mRNAs of crucial zygotic Dorsal target genes were identified. First, activation of target-gene expression expands over time from the ventral-most region of high nuclear Dorsal to lateral regions, where the levels are lower, as a result of a Dorsal-dependent activation probability of transcription sites. Thus, sites that are activated earlier will exhibit more mRNA accumulation. Second, once the sites are activated, the rate of RNA Polymerase II loading is also dependent on Dorsal levels. Morphological restrictions require that translation of the graded mRNA be delayed until completion of embryonic cell formation. Such timing is achieved by large introns, which provide a delay in production of the mature mRNAs. Spatio-temporal regulation of key zygotic genes therefore shapes the pattern of gastrulation.

## Abstract (precis)

**Summary:** Morphogen gradients are known to subdivide a naive cell field into distinct zones of gene expression, but morphogens can also induce a graded response within such domains.

## INTRODUCTION

Morphogen gradients manifest positional information by graded activation of signaling pathways that culminate in the activation of transcription factors. This activation leads to the induction of target genes that establish the fate of the tissue. The most common outcome of morphogen gradients is the division of a target tissue into distinct zones of gene expression, as originally proposed by the ‘French flag’ model (Rogers and Schier, 2011; Wolpert, 1969). The genes triggered within each zone respond to the level of the activating transcription factor, such that different sets of genes are induced accordingly (Sagner and Briscoe, 2019).

Although the morphogen dictates the division of a naive field into distinct uniform zones, there may be instances in which additional patterning within these domains is necessary. For example, in response to the morphogen, coordinated cell movement and tissue morphogenesis may require manifestation of the graded positional information. This output is inherently distinct, in that cells within a given zone would respond differently according to the level of the morphogen. To examine whether a morphogen gradient can also elicit patterning within a designated zone, we have investigated zygotic patterning along the dorsoventral (DV) axis of the *Drosophila* embryo, where both aspects are operating.

Polarity along the DV axis of the *Drosophila* embryo is established by the Spaetzle/Toll signaling pathway (Moussian and Roth, 2005). The ligand Spaetzle (Spz) is distributed in a graded manner within the extracellular perivitelline fluid, which surrounds the embryo, with peak levels at the ventral midline (Haskel-Ittah et al., 2012; Rahimi et al., 2019). Following binding by Spz, activation of the Toll receptor leads to nuclear targeting from the embryo cytoplasm of the NFκB-related transcription factor Dorsal, a process that is graded along the DV axis (Roth et al., 1989; Rushlow et al., 1989; Steward, 1989). The Dorsal nuclear-localization gradient then dictates subdivision of the DV axis into three main zones within the blastoderm embryo (Liberman et al., 2009; Moussian and Roth, 2005).

The *Drosophila* embryo is a syncytium during the initial 13 synchronous nuclear division cycles that follow fertilization. The crucial manifestation of zygotic DV axis target-gene expression takes place during the initial 45 min of nuclear cycle (NC) 14, in parallel with embryo cellularization, during which all ∼6000 cortical nuclei are enclosed in individual cells. Live measurements have shown that graded Dorsal nuclear localization is re-established within 10-15 min of the onset of NC 14 (Rahimi et al., 2019).

**Fig. 1.**
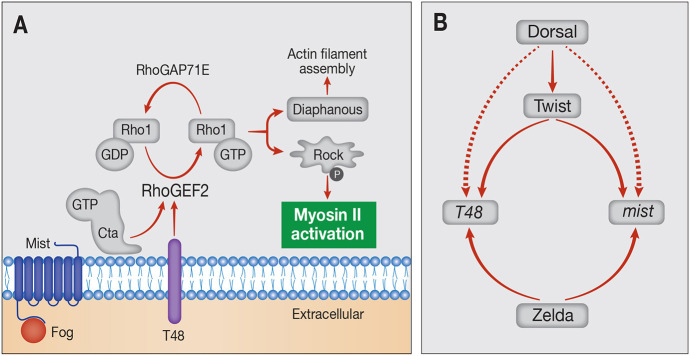
**Transcriptional regulation of zygotic genes driving apical Myosin II recruitment.** (A) Molecular basis of VF formation: Myosin II is recruited and activated apically by Rho1-GTP/Rock, following Rho1 activation by RhoGEF2. Two mechanisms combine to ensure the apical activity of RhoGEF2: extracellular Fog ligand triggers the Mist GPCR to release the Gα protein Concertina (Cta), whereas T48 is an apically localized transmembrane protein that facilitates RhoGEF2 recruitment. All components marked in gray and Myosin II are deposited maternally. Expression of *fog*, *mist* and *T48* is induced zygotically, suggesting that their expression pattern is key to the graded recruitment of Myosin II. (B) Scheme of transcriptional regulation of *T48* and *mist*, encoding transmembrane proteins that function in a cell autonomous manner. Maternal Zld is required to clear nucleosomes of early zygotic genes. Dorsal induces expression of Twi, which, in turn, is essential for *T48* and *mist* expression.

Within the mesoderm, gastrulation involves coordinated three-dimensional cell shape changes and actomyosin-driven movement of mesodermal cells (Fig. S1) (Heer and Martin, 2017; Martin et al., 2009). After completion of cellularization, the ventral cells expressing zygotic *twist* (*twi*) and *snail* (*sna*) invaginate by actomyosin-mediated constriction of their apical surfaces to form the ventral furrow (VF). This constriction is driven by activation of the small GTPase Rho1, which leads to recruitment of the actin nucleator Diaphanous (Dia), as well as Rho kinase (Rock; also known as Rok), which activates myosin II (Fig. 1A). VF formation initiates as a wedge-shaped internalization of the ventral-most cells of the domain, a pattern that results from the graded (ventral to lateral) apical recruitment of myosin (Denk-Lobnig et al., 2021; Heer and Martin, 2017). In mutant backgrounds in which apical myosin recruitment is uniform, the entire cell cohort invaginates simultaneously. Thus, it is necessary to control gene expression within the ventral domain in a manner that will lead to graded myosin recruitment.

Most of the proteins comprising the contractile machinery are provided maternally and, therefore, are ubiquitous and uniformly distributed within the early embryo. Thus, the ability of the Dorsal-nuclear gradient to shape the spatial pattern of mesoderm internalization relies on directing the patterned expression of zygotic target genes that will drive actomyosin contractility. Three key zygotic target genes that are expressed in the ventral part of the embryo, *folded gastrulation* (*fog*), *mist* (also known as *mthl1*) and *T48*, are involved in myosin recruitment. They trigger the key initiating event, activation of Rho1, via its activator RhoGEF2 (Mason et al., 2016). Fog is a secreted ligand that activates G protein-coupled receptors (GPCRs), and Mist is the corresponding GPCR. Binding of Fog to Mist in ventral cells results in the activation and release of the membrane-tethered Gα protein Concertina, leading, in turn, to recruitment from the cytoplasm and activation of RhoGEF2 (Manning et al., 2013; Perez-Vale and Peifer, 2020). In addition, the transmembrane protein T48, which is localized on the apical membrane, mediates RhoGEF2 recruitment to this cellular domain (Kolsch et al., 2007), where myosin II is subsequently required for apical cell constriction (Fig. 1A; Fig. S1).

We examined the impact of the Dorsal-nuclear gradient on the endogenous expression of the zygotic transmembrane regulators of actomyosin contractility, *T48* and *mist* (Fig. 1B). We found that the Dorsal gradient leads to a corresponding graded accumulation of mRNA for both genes through two complementary mechanisms. Activation of gene expression expands over time from the ventral region with high nuclear Dorsal to lateral regions, where the levels are lower. Thus, nuclear Dorsal levels dictate the duration of gene activation. In addition, once a transcription site is activated, the rate of RNA Polymerase II (Pol II, also known as Polr2F) loading depends on the level of nuclear Dorsal. A combination of these two responses contributes to the graded accumulation of *T48* and *mist* mRNA in the cytoplasm. In addition to the expression pattern of these two regulators, the timing of their activity is also crucial because the cell movements of gastrulation should begin only after the completion of cellularization. We found that such timing is dictated by long introns in *T48* and *fog*, which delay the appearance of mature mRNAs that can undergo translation.

## RESULTS

### Activation of *twist* expression follows a switch-like mechanism

*twi*, encoding a bHLH transcription factor, is one of the two cardinal zygotic genes (along with *sna*) that define the ventral mesodermal zone downstream of the Dorsal gradient and subsequently drive the expression of a plethora of tissue-specific genes. To monitor the expression of *twi* quantitatively, we used single-molecule fluorescence *in situ* hybridization (smFISH), which allows visualization of the active transcription sites (TSs) in the nucleus, as well as the accumulation of individual mRNA molecules in the cytoplasm. Given that the analysis is carried out on fixed embryos, each embryo represents a single time point, and the combined analysis of such ‘snapshots’ of multiple staged embryos allows for the reconstruction of the dynamic expression profile.

**Fig. 2.**
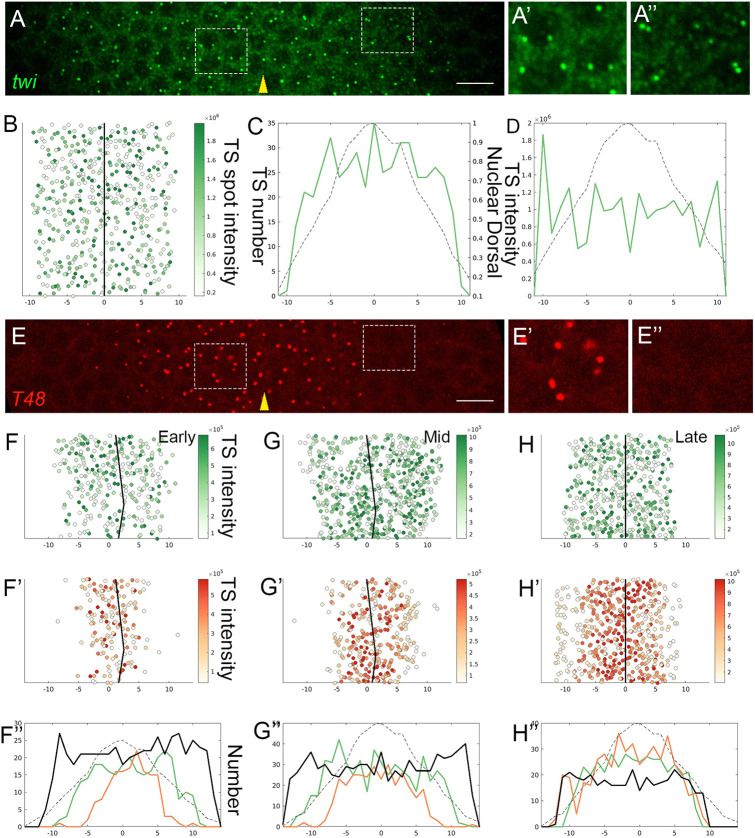
**Dynamics of *twi* and *T48* transcription activation.** (A-A″) *twi* expression in early NC 14 embryos was monitored by smFISH (green), and TSs were readily identified as prominent spots within the nuclei. Prominent *twi* expression was detected in all 20 nuclear columns comprising the future mesoderm. Most nuclei exhibited two TSs per nucleus. The ventral midline is marked by a yellow arrowhead. Magnified views of a ventral and more lateral area within the mesoderm, marked by squares, are shown in A′ and A″, respectively. (B) Quantitative measurement of the intensity of single *twi* TSs within the mesoderm of the embryo visualized in A. In this and all subsequent figures, 0 on the *x*-axis marks the ventral midline, and numbers correspond to nuclear columns. The solid black line represents the ventral midline. *n* (TS number)=480. (C) The number of *twi* TSs per column along the mesoderm is similar. In this and all subsequent figures, the dashed line marks the relative measured amount of nuclear Dorsal according to Ambrosi et al. (2014). (D) The relative median intensity of *twi* TSs is similar along the entire mesoderm domain. These results indicate that *twi* expression is activated by a switch mechanism, at a uniform level throughout the future mesoderm. (E-E″) Expression of *T48* (red, 5′ probe) was monitored by smFISH in the same embryo shown in A. Magnified views in E′ and E″ correspond to the same regions in A′ and A″, respectively. Whereas both *twi* and *T48* TSs are observed in the ventral domain, the lateral mesoderm is devoid of *T48* TSs. These results indicate that Twi is not sufficient for inducing *T48* expression. (F-H″) Monitoring embryos of different ages (as defined by the number of *T48* TSs) within early NC 14 revealed the dynamic expansion of *T48* activation, eventually covering the entire *twi* domain. Shown are quantifications of *twi* TS intensities (F,G,H) and *T48* TS intensities (F′,G′,H′) for three embryos. F″-H″ compare the number of TSs per column of *twi* (solid green line) and *T48* (solid orange line) in these embryos. The solid black line denotes total nuclei number, monitored by DAPI staining. F, *n*=283; F′, *n*=155; G, *n*=565; G′, *n*=321; H, *n*=414; H′, *n*=471. Scale bars: 10 µm.

We found that *twi* expression displays a switch-like behavior, manifesting a sharp on/off threshold in response to Dorsal-nuclear localization levels. Active *twi* TSs appeared simultaneously throughout the entire mesoderm region. The spatial distribution of these sites within the mesoderm was uniform along the DV axis (Fig. 2A-C). Most of the mesodermal nuclei displayed two TSs, indicating that both alleles are transcriptionally active. The median fluorescence intensity of *twi* TSs along the DV axis was also uniform, indicating a similar rate of Pol II loading onto all *twi* loci throughout the mesodermal domain (Fig. 2D). The uniform activation of *twi* transcription is also corroborated by a recent study that followed live accumulation of Twi and identified an early (within ∼5 min) and even appearance of Twi throughout the mesoderm during NC 14 (Dufourt et al., 2021). The rapid activation of *twi* expression by Dorsal could be facilitated by the presence of multiple binding sites for the Zelda (Zld) pioneer factor, which clears the regulatory region from nucleosomes (Sun et al., 2015) (Fig. S2). Moreover, binding of Zld to the *twi* regulatory region is initiated as early as nuclear cycles 8 to 9 (Harrison et al., 2011). In addition, binding of Twi to its own regulatory region (Jiang and Levine, 1993; Zeitlinger et al., 2007) may represent a ‘feed forward’ mechanism, which enhances robust *twi* expression in nuclei in which the levels of Dorsal are above a designated threshold.

### Triggering graded *T48* expression

To examine the transcriptional basis for graded actomyosin contractility within the mesoderm, we followed the expression patterns of *T48* and *mist*, key zygotic target genes that encode transmembrane proteins involved in eliciting a cell autonomous actomyosin response. We followed the expression of *T48* and *twi* in parallel in the same embryos. Similar to *twi*, the *T48* regulatory region also contains multiple sites bound by Dorsal (Sun et al., 2015) (Fig. S2). However, whereas *twi* expression encompassed the entire mesoderm from the outset, the expression of *T48* was initially observed only in the central (ventral-most) part of the mesoderm (Fig. 2E). By contrast, in older embryos, the distribution of TSs for *twi* and *T48* showed a complete overlap (Fig. 2F-H″). We conclude that, whereas *twi* expression is triggered simultaneously in all mesodermal nuclei, the onset of *T48* expression expands over time. A similar temporal expansion has also been observed in live imaging of a MS2-tagged *T48* reporter (Keller et al., 2020; Lim et al., 2017).

Twi is necessary for activation of *T48* expression, because no transcription of *T48* takes place in *twi*-mutant embryos (Kolsch et al., 2007; Leptin, 1991). However, the distinct temporal profiles of *twi* and *T48* expression imply that additional factors besides Twi regulate *T48* expression. The most likely candidate for such a factor is Dorsal, which exhibits graded nuclear levels within the mesoderm. Binding sites for both Twi and Dorsal have been identified on the *T48* enhancer (Zeitlinger et al., 2007; J. Zeitlinger, personal communication). In addition, prominent binding of Zld to the *T48* regulatory region occurred during nuclear cycles 13 to 14 (Harrison et al., 2011) (Fig. S2).

**Fig. 3.**
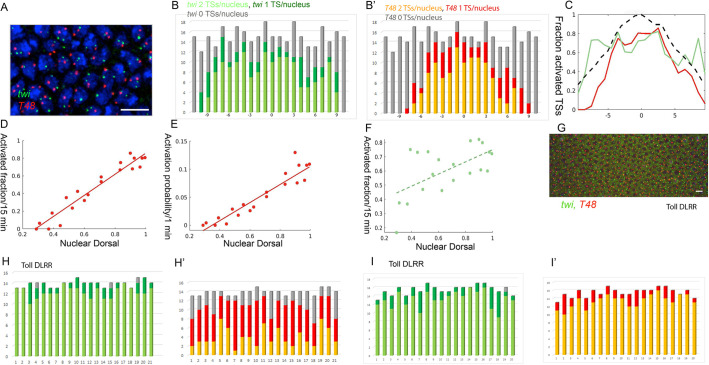
**The probability of *T48* TS activation depends on Dorsal levels.** (A-C) Quantification and graphical analysis of TSs in an early NC 14 embryo probed for *twi* (green) and *T48* (red). Nuclei (A) visualized with DAPI (blue). The probability of TS activation was gauged from the number of TSs activated in each nucleus. Whereas most nuclei within the mesoderm exhibited two *twi* TSs per nucleus (i.e. fully activated), a gradient of *T48* activation was apparent. Most of the ventral nuclei displayed two TSs, whereas the lateral nuclei showed one or no TSs. Identification of many nuclei with only one *T48* TS indicates that the probability of activation of transcription operates independently for each of the two loci within one nucleus. Dashed line shows the relative measured amount of nuclear Dorsal. 0 on the *x*-axis marks the ventral midline, and numbers correspond to nuclear columns. (D) The activated fraction of *T48* loci is linearly correlated with the nuclear Dorsal level (Pearson's r=0.96). (E) The embryo was estimated to be exposed to a stable Dorsal gradient for ∼15 min within NC 14. This time window can be used to calculate the probability of TS activation along the future mesoderm, which is again linearly correlated with the level of nuclear Dorsal (Pearson's r=0.94). (F) In contrast, the correlation between the activated fraction of *twi* loci and nuclear Dorsal levels is significantly weaker (Pearson's r=0.59, dashed line shows linear fit). (G) Uniform expression of the constitutively active Toll^ΔLRR^ construct drives activation of the pathway and nuclear targeting of Dorsal along the entire embryo circumference. Induction of *twi* (green) and *T48* (red) TSs responds accordingly. In a younger embryo (H,H′), two *twi* TSs are observed in most nuclei, whereas only a single *T48* TS is observed in a large fraction of nuclei. In an older embryo (I,I′), the majority of nuclei exhibit two TSs for both genes. Scale bars: 10 µm.

From our analysis of the number of *T48* TSs that were activated in each nucleus at different time points, we concluded that the graded activation reflected the independent response of individual *T48* loci, because many nuclei displayed only one active TS. In other words, although all loci may be primed for expression by binding of maternal Zld, there is a separate probability, dependent on the level of nuclear Dorsal, of activating each *T48* locus to make it permanently accessible for the recruitment of transcription factors and Pol II. Over time, nuclei started to display two active TSs, first in the ventral region and subsequently in more lateral domains. This pattern was distinct from that of *twi* TSs, which mainly occurred as two per nucleus throughout the future mesoderm (Fig. 3A-C). Similar to other early developmental genes, *twi* and *T48* displayed prominent pausing of Pol II prior to transcription activation (Fig. S3) (Saunders et al., 2013). The initial activation event of the transcription start site should entail the release of paused Pol II before the recruitment of subsequent Pol II complexes.

The embryo in Fig. 3A-C is ∼25 min into cycle 14, as estimated from the size and shape of the nuclei (Lecuit and Wieschaus, 2000). Given that establishing a graded pattern of nuclear Dorsal requires 10-15 min following the onset of NC 14 (Rahimi et al., 2019), the nuclei of this embryo were exposed to the Dorsal gradient for ∼15 min. The fraction of nuclei displaying activated *T48* TSs was measured along the ventral domain of this embryo. This value was then used to derive the activation probability per minute at different positions along the DV axis (Fig. 3D,E).

Using this approach, we found that the probability of *T48* TS activation peaked at the ventral midline and declined towards the lateral region. The probability distribution resembles the shape of the Dorsal-nuclear gradient. This tight correlation (Pearson's r=0.94) indicates that the probability of TS activation declined linearly with the reduction in Dorsal levels. Such a monotonic response to Dorsal is in sharp contrast to the nonlinear response of the *twi* enhancer to Dorsal, which generates the sharp borders of *twi* transcription. The activation probability of *twi* expression was indeed less correlated with Dorsal nuclear levels (Fig. 3F).

The instructive role of nuclear Dorsal levels in determining the probability of triggering the *T48* loci was examined in embryos expressing the constitutively active Toll^ΔLRR^ construct, resulting in uniformly high levels of nuclear Dorsal along the length of the DV axis (Rahimi et al., 2016). Nuclei around the entire embryo circumference exhibited a similar response (Fig. 3G). In younger embryos, two *twi* TSs per nucleus appeared in most nuclei, whereas mostly one or (in a minority) two *T48* TSs were observed (Fig. 3H,H′). This intermediate response of *T48* TSs reflects the probability of activation, which requires some time to result in a significant fraction of active TSs. Indeed, in older Toll^ΔLRR^ embryos, most nuclei presented two active TSs for both *twi* and *T48* (Fig. 3I,I′).

### Graded recruitment of Pol II to *T48*

**Fig. 4.**
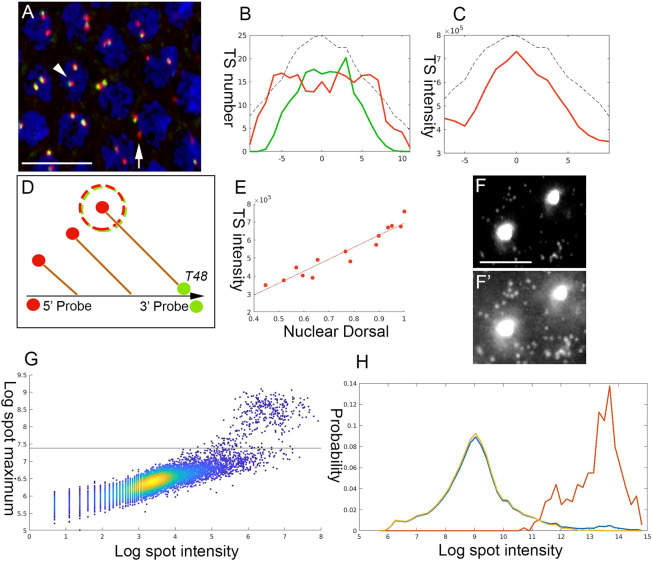
**The rate of Pol II loading on *T48* depends on Dorsal levels.** (A) A region within the mesodermal domain of an early NC 14 embryo hybridized to two distinct *T48* smFISH probes directed against either the *T48* 5′ region (red) or the *T48* 3′ domain (green). TSs with initiated transcription that has not yet reached the 3′ end appear as red dots (arrow), whereas TSs that are producing mature transcripts are yellow (arrowhead). Nuclei are visualized with DAPI (blue). (B) Quantification of TS numbers in this embryo reveals that T48 transcription has been initiated to a similar extent throughout the mesoderm (red) and that Pol II has not yet reached the 3′ end of the gene in a significant number of TSs in the lateral region (green). In B and C, 0 on the *x*-axis marks the ventral midline, numbers correspond to nuclear columns, and the dashed black line represents the relative measured amount of nuclear Dorsal. (C,D) The *T48* Pol II loading capacity along the mesoderm was followed by monitoring the median intensity of the 5′ probe (red) only in those TSs that also exhibited a signal for the 3′ probe (dashed circle in D). (E) A distinct dependence of the loading rate on the position along the DV axis (and, hence, Dorsal level) was observed (Pearson's r=0.47). (F,F′) *T48* signal intensity was followed with a smFISH probe covering the entire coding sequence (gray). The prominent spots in the nuclei represent the TSs, whereas the weak spots in the cytoplasm correspond to individual mRNA molecules. Different levels of exposure of the same image highlight the two types of signal. (G) The maximal pixel (*y*-axis) and the cumulative pixel intensity (*x*-axis) of each spot were compared. The rationale is that single or cytoplasmic clusters of mRNA molecules would be present in large numbers and display a lower maximal intensity because of their lower concentration. The TSs would be marked by high maximal intensity because of the elevated local concentration of mRNA molecules. The horizontal black line distinguishes the two populations. (H) Cumulative spot intensity histogram identifies two peaks, corresponding to the median single mRNA intensity and TS intensity, respectively (total number, blue; mRNA, yellow; TS, red). The median intensity of the TSs was 70 times higher than that of the single mRNA, providing an estimate of the average *T48* Pol II loading. Taking the size of *T48* into account, this value is ∼ten-fold lower than the maximal Pol II loading capacity, indicating that differential Pol II loading is likely within the linear range. Scale bars: 10 µm in A; 2 µm in F,F′.

Median intensity of the individual *twi* TSs across the mesoderm appeared constant (Fig. 2D), indicating a comparable rate of Pol II loading. Measuring and analyzing the intensity of *T48* TSs as a proxy for the number of polymerases traversing the gene is more complex, given the gradual, time-dependent progression of expression initiation of this gene along the DV axis. Therefore, it is imperative to only consider those *T48* loci that are already at a steady-state and are producing mature transcripts. Thus, we used distinct smFISH probes for the 5′ and 3′ domains of *T48* and limited our *T48* TS intensity analysis to loci that displayed both a 5′ and a 3′ probe signal, indicating that some Pol II molecules had reached the 3′ region of the gene (Fig. 4A-D). Monitoring the intensity of the 5′ probe for these sites provided a cumulative measure of the number of polymerases along the gene. A graded median intensity gradient was observed across the ventral region (Fig. 4C), with a tight correlation of the Pol II loading rate with the position of nuclei (and, hence, with nuclear Dorsal levels) (Pearson's r=0.47) (Fig. 4E).

To determine the average number of Pol II complexes that are loaded on *T48*, we compared the intensity of nuclear TSs with that of individual mRNA spots in the cytoplasm, using a probe covering the entire coding sequence (Fig. 4F). The cumulative and maximal pixel intensity and volume of each spot were monitored. The rationale for this approach was that the cumulative spot intensity indicates the number of mRNAs, whereas the maximal intensity distinguishes highly concentrated mRNA molecules at the TS in the nuclei from the more-dispersed mRNA clusters in the cytoplasm. A histogram across the spot intensity (in Log scale) for each of these groups identified two peaks, corresponding to the median intensity of a single mRNA and the TS, respectively (Fig. 4G,H). The median intensity of the TSs was 70 times higher than that of the single mRNAs, providing an estimate of the average extent of Pol II loading onto *T48* loci (Fig. 4G,H). Given that *T48* is 29 kb long, this translates to a density of one Pol II complex every ∼400 bp, which is about tenfold lower than the maximal loading that is physically possible. Thus, control of the number of Pol II molecules loaded on the *T48* locus is likely within the linear dynamic range.

### Dual mechanism leading to graded *T48* mRNA accumulation

**Fig. 5.**
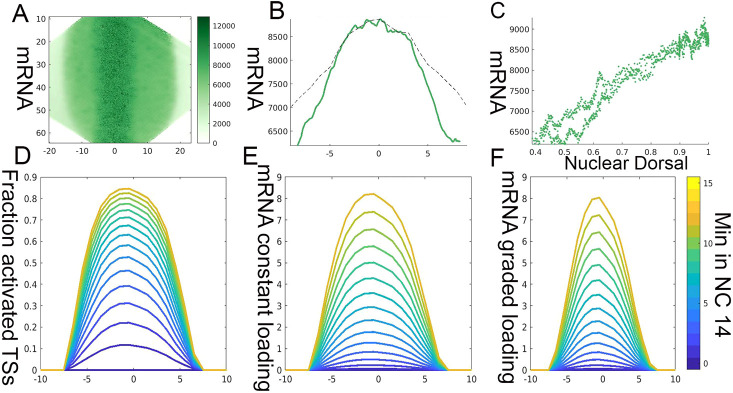
**Activation probability and graded Pol II loading generate graded *T48* mRNA accumulation.** (A,B) To follow the pattern of *T48* mRNA cytoplasmic accumulation and omit TS spots, the median pixel intensity at each distance from the midline was calculated for an embryo in which *T48* TSs could be observed at the lateral edges of the mesoderm. A graded accumulation of *T48* mRNA along the future mesoderm is readily apparent and is correlated with the levels of nuclear Dorsal. (C) The level of cytoplasmic *T48* mRNA correlates with the distance from the ventral midline and nuclear Dorsal (Pearson's r=0.95). (D) Based on the probability of *T48* TS activation that was calculated from experimental data (Fig. 3), the fraction of activated nuclei over time was simulated. (E) If we assume that the rate of Pol II loading onto activated gene loci is similar throughout the mesoderm, a gradient of mRNA accumulation will still be generated, because ventral loci are activated earlier than more lateral ones. (F) The resulting mRNA gradient sharpens further when we include a graded Pol II loading rate in the simulation. The combined effect of Dorsal-dependent activation probability and graded loading gives rise to graded accumulation of *T48* transcripts in the cytoplasm. 0 on the *x*-axis in A,B,D-F marks the ventral midline, and numbers correspond to nuclear columns.

A combination of the two mechanisms outlined above (graded onset of TS activation and graded polymerase loading rates of the gene across the ventral region) should give rise to a graded distribution of *T48* mRNA. Indeed, quantitation of *T48* mRNA in the cytoplasm showed a graded pattern that was highly correlated with nuclear Dorsal levels (Pearson's r=0.92±0.02) (Fig. 5A-C). Given that Twi distribution in the mesoderm was uniform, we assume that nuclear Dorsal underlies the graded expression of *T48*.

The quantitative measurement of *T48* TS activation (Fig. 3D,E) allows simulation of the dynamic pattern of production and accumulation of mRNA (Fig. S4). The fraction of activated TSs increases over time in a graded manner. If every activated site is transcribing at a similar rate, this would still lead to the graded accumulation of mRNA, because the ventral TSs were activated earlier (Fig. 5D,E). We assume that the transcribed mRNAs are stable within the relevant time window.

In addition to the temporal order of TS activation, the actual rate of Pol II loading depends on the level of nuclear Dorsal. When this aspect was added to the simulation, the shape of the accumulating mRNA peak became sharper (Fig. 5F). Given that the activation of TSs reached saturation at the midline (i.e. almost all TSs become activated), this additional feature contributes to the shape of the mRNA peak within the relevant time window.

### Graded accumulation of *mist* transcripts

**Fig. 6.**
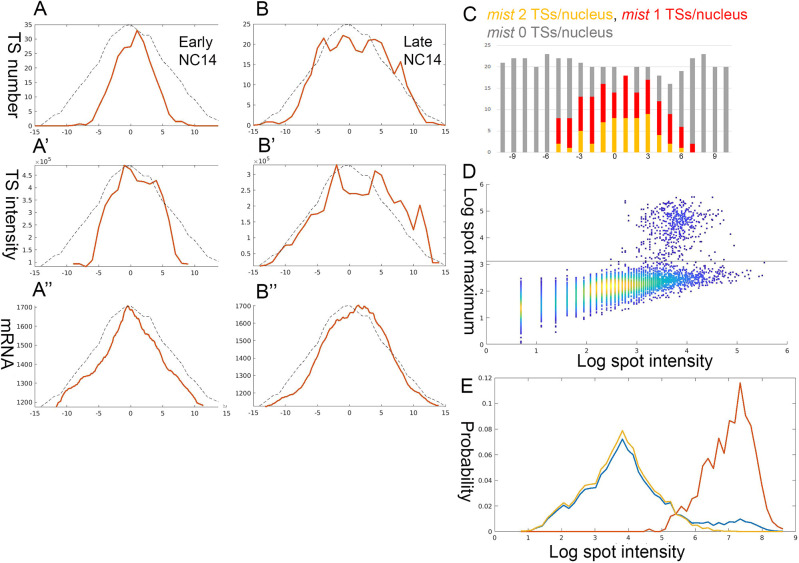
**Graded accumulation of *mist* transcripts.** Quantification and graphical analysis of *mist* expression during early NC 14 was performed using a single 5′ region smFISH probe. (A-B″) Expression of *mist* in a pair of embryos displays similar hallmarks to those identified for *T48*. Dynamic *mist* activation was seen by the lateral expansion of TSs over time (the embryo analyzed in A-A″ is younger than that analyzed in B-B″). Pol II loading depends on Dorsal levels, as indicated by the graded TS intensity (Pearson's r=0.21). The resulting mRNA accumulation of *mist* displays a sharp graded pattern (Pearson's r=0.95). (C) The Dorsal-dependent activation probability of *mist* can be assessed by monitoring the number of activated loci per nucleus. 0 on the *x*-axis in A-C marks the ventral midline, and numbers correspond to nuclear columns. (D,E) The median number of Pol II complexes per *mist* locus was calculated as 30. Taking the gene size into account, this corresponds to an average density that is 1.4 times higher than that calculated for *T48* but is still well within the linear range.

Expression of *mist* was analyzed in a similar way to that of *T48* and exhibited comparable features. Binding by Twi, Dorsal and Zld has been identified on the *mist* enhancer (Sun et al., 2015) (Fig. S2). The *mist* enhancer also displayed paused Pol II (Fig. S3) (Saunders et al., 2013). Graded activation of TSs was monitored over time, with the initial activation taking place along the ventral midline (Fig. 6A,B). A gradient of *mist* TS intensities, reflecting a position-dependent Pol II loading rate, was apparent (Fig. 6A′,B′). Of note, because *mist* is a relatively short gene (9.3 kb), we assumed that transcription had reached a steady-state on all TSs in older embryos. *mist* mRNA accumulated in the cytoplasm in a graded manner (Fig. 6A″,B″). Monitoring the number of activated TSs per nucleus revealed more nuclei with two TSs in the ventral region (Fig. 6C). Finally, uniform nuclear targeting of Dorsal following expression of Toll^ΔLRR^ resulted in a uniform level of *mist* activation along the DV axis (Fig. S5).

To determine the Pol II loading rate onto the *mist* loci, the intensity of TSs and single mRNA spots was compared (Fig. 6D,E). From this, a median number of ∼30 Pol II complexes per gene was estimated. Taking the size of the gene (9.3 kb) into account, this distribution was 1.4 times denser than that calculated for *T48*, but still well below saturation levels.

### Intron delay of *T48* expression

**Fig. 7.**
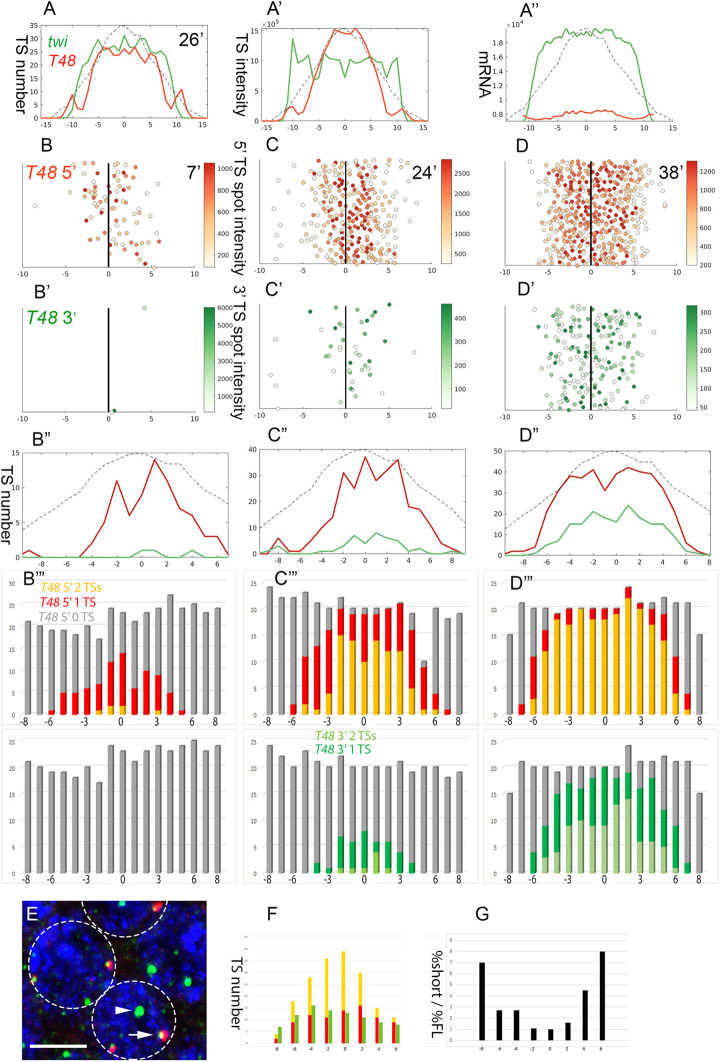
**Intron delay dictates the timing of *T48* translation.** (A-A″) Expression of *twi* (green) and *T48* (red) was monitored simultaneously in the embryo analyzed in Fig. 2, which is estimated to be 26 min into NC 14. *twi* mRNA accumulation was prominent. Although not all nuclei exhibit *T48* TSs at this stage, the TSs at the ventral region were prominent. However, no *T48* mRNA was detected. We hypothesized that the unusually large intron of *T48* may account for the delay in transcript maturation. (B-B‴) An embryo 7 min into NC 14 displayed the onset of *T48* 5′ transcription (red) in the ventral-most nuclei (*n*=78) mostly as a single TS/nucleus, but no 3′ signal (green) was detected (*n*=3). (C-C‴) By 24 min into NC 14, the 5′-positive TSs expanded laterally (*n*=278), with more nuclei showing two TSs, whereas the 3′-positive TSs were only beginning to appear (*n*=43). (D-D‴) By 38 min, both probes expanded laterally (5′ *n*=428, 3′ *n*=182), and mRNA in the cytoplasm was evident. (E) To directly assess the contribution of the intron, a *T48* cDNA transgene that is driven by the *T48* enhancer was generated. Given that the transgene is inserted at a different chromosomal position, it should display a TS signal distinct from the endogenous *T48* loci. To distinguish between the endogenous and cDNA transcripts, embryos were probed with a *T48* intron probe (red) and 3′ probe (green). We expect the endogenous TSs to be marked by both probes (arrow), whereas the cDNA should only react with the 3′ probe (arrowhead). Nuclei are marked by DAPI (blue). (F) The number of mature endogenous TSs (yellow) peaks at the ventral midline of an intermediate stage embryo, whereas the lateral region harbors transcripts that have reached the intron but not the 3′ end (red). In contrast, a fairly uniform distribution of cDNA TSs was observed (green), indicating that the cDNA TSs matured earlier than the endogenous loci. (G) Plotting the ratio between the relative fraction of mature cDNA versus endogenous TSs along the mesoderm demonstrates the faster maturation of the cDNA TSs. 0 on the *x*-axis marks the ventral midline, and numbers correspond to nuclear columns. The solid black line represents the ventral midline. Scale bar: 5 µm.

The graded accumulation of *T48* and *mist* mRNAs is effectively driven early in NC 14, following completion of the final syncytial cycle of nuclear divisions. However, the protein products that trigger apical actomyosin contractility, including RhoGEF2 (Mason et al., 2016) and Myosin II (Dawes-Hoang et al., 2005), are recruited apically and function only after cellularization is complete, 30-40 min after the onset of NC 14 (see also Movie 1), necessitating a delay. An indication that regulation of mature mRNA production may contribute to this timing mechanism was observed when comparing the cytoplasmic mRNA accumulation patterns of *twi* and *T48*. As reported above (Fig. 2), early NC 14 embryos displayed prominent, active TSs for both *twi* and *T48*. However, whereas *twi* mRNA was readily detected in the cytoplasm of such embryos, no *T48* mRNA was observed in an embryo that was ∼26 min into NC 14 (embryo age was determined throughout from the apical-basal length of DAPI-stained nuclei) (Lecuit and Wieschaus, 2000) (Fig. 7A-A″; Fig. S6). Given that *twi* is a short gene of 2.2 kb, no delay in completion of transcription is expected to take place. In contrast, *T48* is 29 kb long, because of the presence of large introns encompassing ∼25 kb. This organization is exceptional for early zygotic genes, which are typically intron-less or have only short introns (Artieri and Fraser, 2014). Given a transcription rate of ∼2 kb/min (Fukaya et al., 2017), such long introns could delay the production of mature mRNA and protein until after the completion of cellularization. Interestingly, *fog* also displayed similarly sized large introns. Although the presence of Fog is key for the timing of Mist activation, its distribution may not be instructive because it is secreted to the fluid extracellular milieu.

If the intron prolongs *T48* transcription, using distinct *T48* 5′ and 3′ probes we should expect temporal and spatial differences (because of the expansion of TS activation) in the detection of TSs. The onset of *T48* 5′ transcription was observed in the ventral-most nuclei by 7 min into NC 14, with no apparent detection of TSs displaying the 3′ probe. The positive nuclei predominantly displayed a single 5′ TS (Fig. 7B-B‴; Fig. S6). By 24 min, 5′-probe-displaying TSs expanded laterally and more nuclei displaying two spots appeared. TSs that also showed the 3′ probe began to appear in the ventral-most nuclei (Fig. 7C-C‴; Fig. S6). The ∼17 min delay between the two panels provides an estimate of the time required for Pol II to complete *T48* transcription at 20°C. By 38 min, TSs displaying both 5′ and 3′ probes were detected throughout the mesoderm, with most nuclei showing two spots (Fig. 7D-D‴; Fig. S6). *T48* mRNA was then observed by both probes in the cytoplasm.

If the delay is caused by transcription through the *T48* intron, then transcription of intron-less *T48* within a given nucleus should be completed earlier than that of the endogenous locus. To test this prediction, we fused a *T48* cDNA construct to its 5′ regulatory region (Lim et al., 2017) and generated transgenic flies that integrated the construct at a locus that was different from the endogenous one. By using distinct intron and 3′ probes, we could distinguish the completion of transcription at endogenous loci by their reactivity with both probes, whereas the cDNA locus reacted only with the 3′ probe (Fig. 7E). Analysis of the distribution of both TS types demonstrated that completion of transcription for the cDNA TSs occurred earlier than that of the endogenous *T48* locus. Whereas the mature endogenous TSs were detected mostly in the ventral domain, TSs exhibiting the cDNA 3′ domain were distributed approximately uniformly throughout the mesoderm (Fig. 7F). Accordingly, the fraction of cDNA TSs was higher at the lateral domain (Fig. 7G).

## DISCUSSION

### Graded response to a morphogen gradient

The primary hallmark of pattern formation based on morphogen gradients is the ability to use a graded morphogen distribution for the induction of distinct zones of gene expression. We explored whether the information conveyed by the morphogen gradient can also be used to generate a graded response within defined domains of the target tissue. In situations in which the morphogen gradient is the only symmetry-breaking cue, morphogenetic movements of distinct zones in the target tissue may also require a graded transcriptional response to the morphogen.

We examined this paradigm during the establishment of the DV axis in the *Drosophila* embryo, in which the gradient of nuclear localization of the Dorsal protein represents the only asymmetric cue for patterning of the embryo along this axis. The future mesoderm undergoes graded constriction by an actomyosin network in order to fold and internalize in a wedge-shaped pattern. If this behavior is guided by a zygotic mechanism in the embryo, its origins may well lie in a mesoderm-specific transcriptional response to the Dorsal nuclear gradient.

To examine the underlying transcriptional mechanism for graded actomyosin constriction, we focused on two zygotic genes encoding transmembrane proteins that regulate the activation of the small GTPase Rho1, a key initiating event in the process. T48 was shown to recruit RhoGEF2 to the apical surface (Kolsch et al., 2007), and Mist is a GPCR that is activated apically by the ligand Fog, releasing the Gα protein Concertina, which activates RhoGEF2 (Manning et al., 2013; Mason et al., 2016; Perez-Vale and Peifer, 2020) (Fig. 1).

We found two distinct Dorsal-dependent mechanisms that generate graded mRNA accumulation for *T48* and *mist*. Initially, expression of both genes was triggered only in the central (ventral-most) domain of the mesoderm, expanding laterally over time. This expansion was consistent with a TS activation response to the Dorsal nuclear gradient, which is maximal at the ventral midline. Distribution of the number of activated TSs per nucleus indicated that the probability of activation is independent for each allele. Thus, two alleles within the same nucleus that experience similar levels of nuclear Dorsal may be switched on at different times. The probability of TS activation was linearly correlated with the levels of nuclear Dorsal.

The molecular nature of the Dorsal-dependent mechanism that leads to activation of *T48* and *mist* TSs is not known. Transcription of early zygotic genes in the embryo is facilitated by binding of the pioneer factor Zld, which removes nucleosomes from the regulatory regions (Sun et al., 2015). Enrichment of Zld binding to these loci indicates that nucleosomes are cleared from these regulatory regions. Furthermore, enhancement of Zld binding was shown to increase the transcription rate of a *T48* reporter construct (Keller et al., 2020). These enhancers also display binding sites for Twi, which is more prominent and uniform across the mesoderm and, thus, stably associates with the loci across the future mesoderm. Whereas nuclear Dorsal levels are limiting, once Dorsal is recruited to the locus, a stable association between Twi and Dorsal proteins may stabilize Dorsal binding and permanently alter the future accessibility of the site to incoming Pol II. Binding of Dorsal and Twi to target genes, such as *single minded*, was shown to facilitate their transcription by enhancing the probability of DNA binding by the Notch intracellular domain (Falo-Sanjuan et al., 2019).

The different transcription onset times are expected to generate graded mRNA accumulation, with higher levels adjacent to the nuclei that were induced earlier. The effectiveness of this mechanism is restricted to a defined time window. When most of the transcription sites in the ventral region are triggered over time, the differences in mRNA production diminish. Indeed, monitoring apical Myosin II recruitment showed that lateral mesodermal cells eventually recruit similar levels to the ventral-most cells (Bhide et al., 2021). For optimal function, the probability of transcription activation should be high enough to prime a significant number of TSs and yet not plateau within the critical time window.

**Fig. 8.**
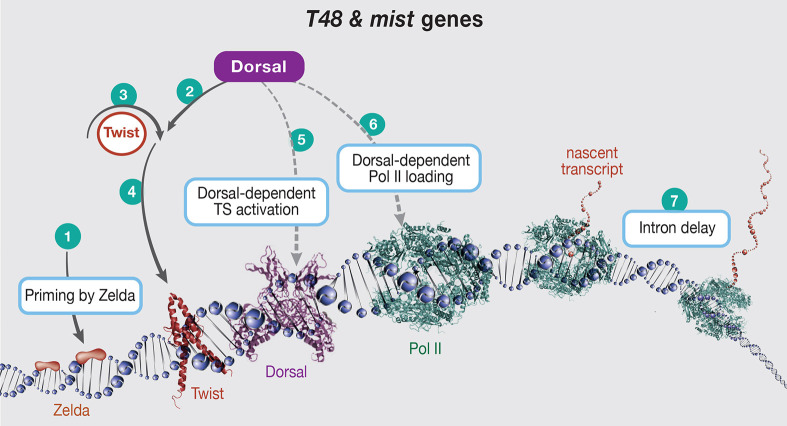
**Graded accumulation of *T48* and *mist* transcripts drives gastrulation.** Schematic of *T48* and *mist*, depicting proposed inputs from DV axis elements and gene transcription machinery, leading to graded mRNA accumulation in the mesoderm. (1) The pioneer factor Zld, which is maternally provided, clears nucleosomes from the regulatory regions of early zygotic genes. (2) The nuclear Dorsal protein gradient is the only source of asymmetry along the DV axis of the early *Drosophila* embryo. Dorsal binding to the *twi* enhancer triggers expression with a sharp threshold. (3,4) The autoregulatory capacity of Twi assures robust and uniform *twi* expression along the future mesoderm. (5) Twi is also essential for *T48* and *mist* expression, but it is not sufficient. In order to trigger expression from these loci, Dorsal is also required. Progressive induction over time depends on Dorsal nuclear levels. The probability of activation is dictated by the limiting levels of Dorsal and, therefore, will progress over time from the ventral to the lateral region. (6) Once activated, loading of Pol II also depends on Dorsal binding. Thus, the transient binding of Dorsal, which is dictated by its nuclear concentration, will modulate the transcription burst size or frequency. A combination of both of these mechanisms will lead to a graded accumulation of mRNA. (7) Finally, generation of mature, translation-ready transcripts of the proteins leading to Myosin II recruitment can be influenced by the intron size of the genes. Unusually long introns in *T48* (and *fog*) yield a delay in transcript maturation, thereby providing a timing mechanism that ensures translation of the proteins only after completion of cellularization.

Once a TS is activated, the rate of Pol II loading is also dependent upon nuclear Dorsal levels. We can imagine a scenario in which binding of Dorsal to the enhancers has on and off rates, such that the fraction of time that Dorsal is bound would depend on its concentration. Analysis of a reporter *T48* construct indicated that Dorsal-dependent Pol II activity is modulated via the on rate, whereas the off rate is fairly constant (Keller et al., 2020). The different Pol II loading rates would also contribute to the generation of an mRNA gradient. Furthermore, this mechanism would continue to operate even after the activation of TSs begins to plateau. Simulations based on our measurements showed that a combination of the two mechanisms can lead to a robust gradient of mRNA accumulation. Graded alteration in transcriptional burst size was also recently demonstrated for BMP target genes in the dorsal ectoderm of the *Drosophila* embryo (Hoppe et al., 2020). However, it is not clear whether this graded response has a biological role in this setting. The proposed mechanisms underlying graded activation of transcription by Dorsal are summarized in Fig. 8.

As for any biological mechanism that employs  probability, the question of the precision and robustness of the outcome arises. Wedge-shaped mesoderm folding relies on relative differences in contractility between the cells, not on absolute levels, such that the system is less restricted. Furthermore, the connectivity between cells, mediated by cell junctions, may average out local perturbations. The tension along the anterior-posterior axis of the embryo was shown to be greater than that along the DV axis, leading to the distinct elongated shape of mesoderm invagination (Chanet et al., 2017). This global tension may also act as a buffering element for local perturbations.

### Timing of actomyosin constriction by intron delay

Timing is a central issue when coordinating the processes of cellularization and mesoderm invagination in *Drosophila* embryos during NC 14. The Dorsal nuclear gradient is stabilized within 10-15 min, shortly after onset of NC 14 (Rahimi et al., 2019). The generation of the mRNA gradient for *T48* and *mist*, encoding proteins regulating actomyosin constriction, takes place early in NC 14, whereas the process of cellularization, which is driven by elongation of the cell membranes towards the basal side, is completed 15-30 min later. Therefore, it is imperative to ensure that the graded apical recruitment of Myosin II driving apical constriction of the gastrulating cells is delayed until this time (Dawes-Hoang et al., 2005), because premature apical constriction before cellularization is completed may be deleterious.

Most early zygotic genes, including those expressed in the future mesoderm, are intron-less or exhibit introns of only a few kb (De Renzis et al., 2007). *T48* and *fog* stand out, displaying introns of 25 kb. This large intron size is conserved in the homologous genes of different *Drosophila* species. Given that the measured speed of Pol II progression is ∼2 kb/min (Fukaya et al., 2017), these introns could provide a crucial delay of 10-15 min in the production of the mature mRNA. We have demonstrated that this delay does indeed take place, by comparing the detection timing of 5′ versus 3′ *T48* probes and by demonstrating that an mRNA produced by an intron-less *T48* matured before the transcript produced from the endogenous locus (Fig. 7). It is interesting that the intron sizes of *T48* and *fog* are similar, facilitating a coordinated timing of mature mRNA appearance. T48 may generate a graded pattern, whereas the production of Fog, a diffusible extracellular ligand, could provide a temporal switch. If the Fog receptor, Mist, is produced in a graded manner, it will lead to graded activation. Given that *mist* is only 9.3 kb long, Mist may appear before the presentation of Fog. The manifestation of intron delay in timing was previously demonstrated for *kni related* (also known as *knirps-like*) in the *Drosophila* embryo (Rothe et al., 1992), and for *her7*/*Hes7* in the segmentation clock in zebrafish and mice (Lewis, 2003; Takashima et al., 2011). Intron length may provide an effective temporal design mechanism in situations in which intricate control of timing is necessary (Swinburne and Silver, 2008).

In conclusion, the Dorsal nuclear gradient is used not only for subdivision of the embryonic DV axis into distinct domains, but also for specification of graded responses leading to tissue shape changes that culminate in gastrulation. The convergence of two distinct mechanisms for generating transcriptional gradients of genes regulating actomyosin contractility sharpens the resulting response. Finally, temporal coordination between the graded transcriptional response and execution of cell movement is achieved by a delayed maturation of the relevant transcripts, dictated by intron size.

## MATERIALS AND METHODS

### Fly lines

The bulk of experiments were performed using wild-type (*yw*) embryos.

For ectopic Toll activation, we analyzed embryos generated by females carrying *UASp-Toll*^Δ*LRR*^ and the *nos-Gal4* driver (Rahimi et al., 2016). Intronless *T48* was generated by fusing the cDNA to the *T48* mesodermal enhancer and the *eve* minimal promoter (Lim et al., 2017). The construct was generated by GenScript, cloned into pATTB and injected into attP40 flies by BestGene. Movie 1 shows the visualization by lightsheet microscopy of a Myosin II (Zipper)-GFP embryo (BDSC line B-51564).

### smFISH

Probe sets were designed by Stellaris Probe Designer and purchased from LGC Biosearch Technologies (see Table S1). Three hours after egg lay (AEL), embryos were fixed for 25 min in 4% formaldehyde, washed in methanol and kept at −20°C. The following day, embryos were washed in methanol and then in ethanol, rocked in 90% xylene:10% ethanol for 1 h, post-fixed in 4% paraformaldehyde/PBS/0.05 M EGTA for 25 min, washed three times (10 min each), then incubated for 6 min with 10 µg/ml Proteinase K recombinant, PCR Grade (Roche, RPROTKSOL-RO) and post-fixed again. Embryos were transferred gradually to 10% Formamide Deionized (FA; Thermo Fisher Scientific; AM9342) in 2× SSC+10 μg/ml ssDNA preheated to 37°C and prehybridized for 30 min at 37°C. Hybridization buffer included 10% FA, 10% dextran, 2 mg/ml bovine serum albumin (Thermo Fisher Scientific; AM2616), ribonucleoside vanadyl complex (RVC; New England Biolabs; S1402) and ssDNA+ tRNA in 2× SSC, containing the probe set (1 ng/μl) (Trcek et al., 2017). Hybridization was carried out overnight at 37°C. Next morning, the embryos were shaken gently and incubated for another 30 min. Embryos were then washed twice for 30 min each at 37°C with 10% FA in 2× SSC+10 μg/ml ssDNA and gradually transferred to PBS-0.5% Tween 20, Molecular Biology Grade (Promega, H5151) and mounted with Vectashield+DAPI Mounting Medium Vectashield+DAPI Mounting Medium (Vector Laboratories, H-1200-10). Fluorescence was visualized with a Zeiss LSM800 confocal microscope or Nikon Eclipse Ti2 microscope (Rahimi et al., 2019).

### Image analysis for TS detection and TS and mRNA quantification

After acquisition, raw images were processed in MATLAB (MathWorks). First, and for each channel, the image stack was maximally projected along the *z*-axis and the TS spots in each channel were then detected using the method of Raj et al. (2008). In brief, *z*-projected images were processed with a log-filter (size=15 px, σ=2.5 px), and the detection threshold was defined as the first threshold in the environment of which the number of detected TSs stayed relatively constant [change (in log) smaller than half the maximal relative change across all thresholds (in log)]. Then, eight-connected pixels above the threshold were defined as distinct TSs and their intensity defined as the sum of their pixel intensities. For each embryo, the midline was then defined according to *twi* fluorescence (or *T48* if *twi* was not available) and the distance (in pixels) between each TS and the midline was calculated. To convert the pixel measurement into nuclei columns, the DAPI image was used to determine the average column distance for each embryo and all distances were converted accordingly. For comparisons with the Dorsal nuclear level, Dorsal levels in each nuclear column were taken from Ambrosi et al. (2014) and rescaled so that the maximal Dorsal level at the midline corresponded to 1. For each embryo, only the TSs (and mRNAs) inside a manually selected region of interest (∼400 nuclei) were analyzed. mRNA levels at each distance from the midline were calculated as the median fluorescence of all pixels with this midline distance. All codes for TS and mRNA analyses can be found on GitHub (https://github.com/barkailab).

### Single mRNA detection and Pol II quantification

After acquisition, raw image stacks were processed in MATLAB. For each fluorescence channel, the ‘local’ background fluorescence (median fluorescence in a moving sphere) was first determined and subtracted from the raw signal. The background-corrected image stacks were processed with a three-dimensional log-filter, and the spots on the processed image stack detected with the mRNAcount function from Raj et al. (2008). For each 18-connected spot (mRNA and TSs), the cumulative and maximal pixel intensities were determined from the fluorescence value of all corresponding pixels. The spots were then classified as mRNA spots (single, cytoplasmic molecules) or TS spots (multiple, transcribed mRNAs at the TS) based on their maximal intensity (Fig. 4A).

### Activation probability calculation and mathematical mRNA expression model

Based on our results, TS activation was assumed to be a stochastic, independent process and, thus, the activated promoter fraction (at a certain nuclear column) can be described by 1−exp(−T*p), with *T* being the time in NC 14 and *p* being the activation probability. This formula was used to calculate the activation probabilities in Fig. 3E. This experimentally derived probability was used as an input for the dynamic model in Fig. 5. In addition, the activation probability outside the midline was assumed to be linearly related to Dorsal levels [as measured by Ambrosi et al. (2014)] and the mRNA production rate after TS activation was assumed to be either constant (=1) between the different TSs or fixed at the midline (=1) and linearly related to the Dorsal levels at other positions. After transcription, mRNAs were assumed to be stable and, thus, to accumulate over time.

### Data analysis

To calculate the correlation between nuclear dorsal level and the activated fraction or activation probability (Fig. 3D-F), we first calculated the activated fraction and activation probability from the number of active TSs in the different cell rows (compare with Fig. 3B). Pearson’s correlation was used to determine the linear correlation between these parameters and the nuclear Dorsal level in the corresponding cell rows, according to Ambrosi et al. (2014). To calculate the correlation between nuclear dorsal level and intensity for individual TSs (Fig. 4E), we first linearly interpolated the Dorsal values of individual TSs (and nuclei) from the row-based measurement (Ambrosi et al., 2014), based on their exact distance from the midline. Pearson’s correlation was then used to calculate the linear correlation between each spot intensity of the TSs and the interpolated nuclear Dorsal level. For visualization, the median TS intensity in the different cell rows and its fit to the nuclear Dorsal level is shown.

## Supplementary Material

Supplementary information

Reviewer comments
