# Supplementary material for: Generation and timing of graded responses to morphogen gradients
Source: Development. 2021 Dec 17;148(24):dev199991. doi: 10.1242/dev.199991 (PMC8722393; doi:10.1242/dev.199991)
Supplement: Supplementary information [file develop-148-199991-s1.pdf]

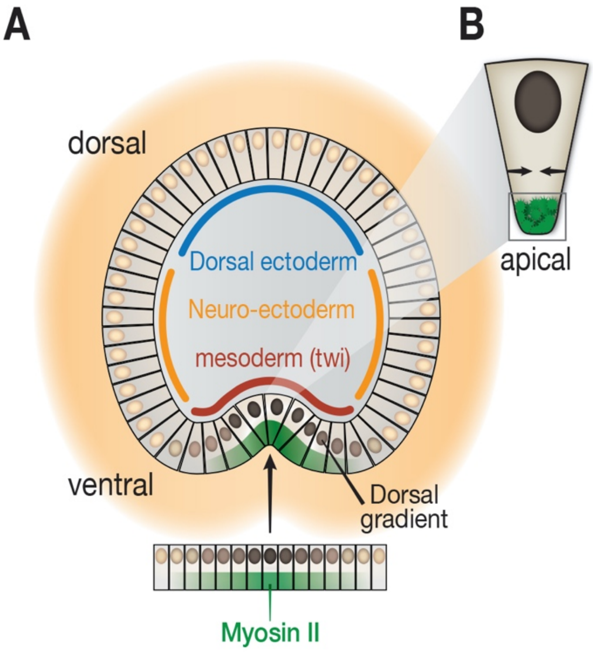

Fig. S1. Graded recruitment of Myosin II drives gastrulation
A) Schematic cross-section of an embryo initiating ventral furrow (VF) invagination during NC 14, after completion of cellularization. The nuclear gradient of Dorsal protein drives expression of *twist* in the mesoderm, and subdivides the embryo into three zones of gene expression. Recruitment of Myosin II (green shading) to the apical surface of the ventral cells drives their apical constriction and invagination. B) The apical wedge-shape of the gastrulating tissue is triggered by graded recruitment of Myosin II, peaking at the ventral midline.

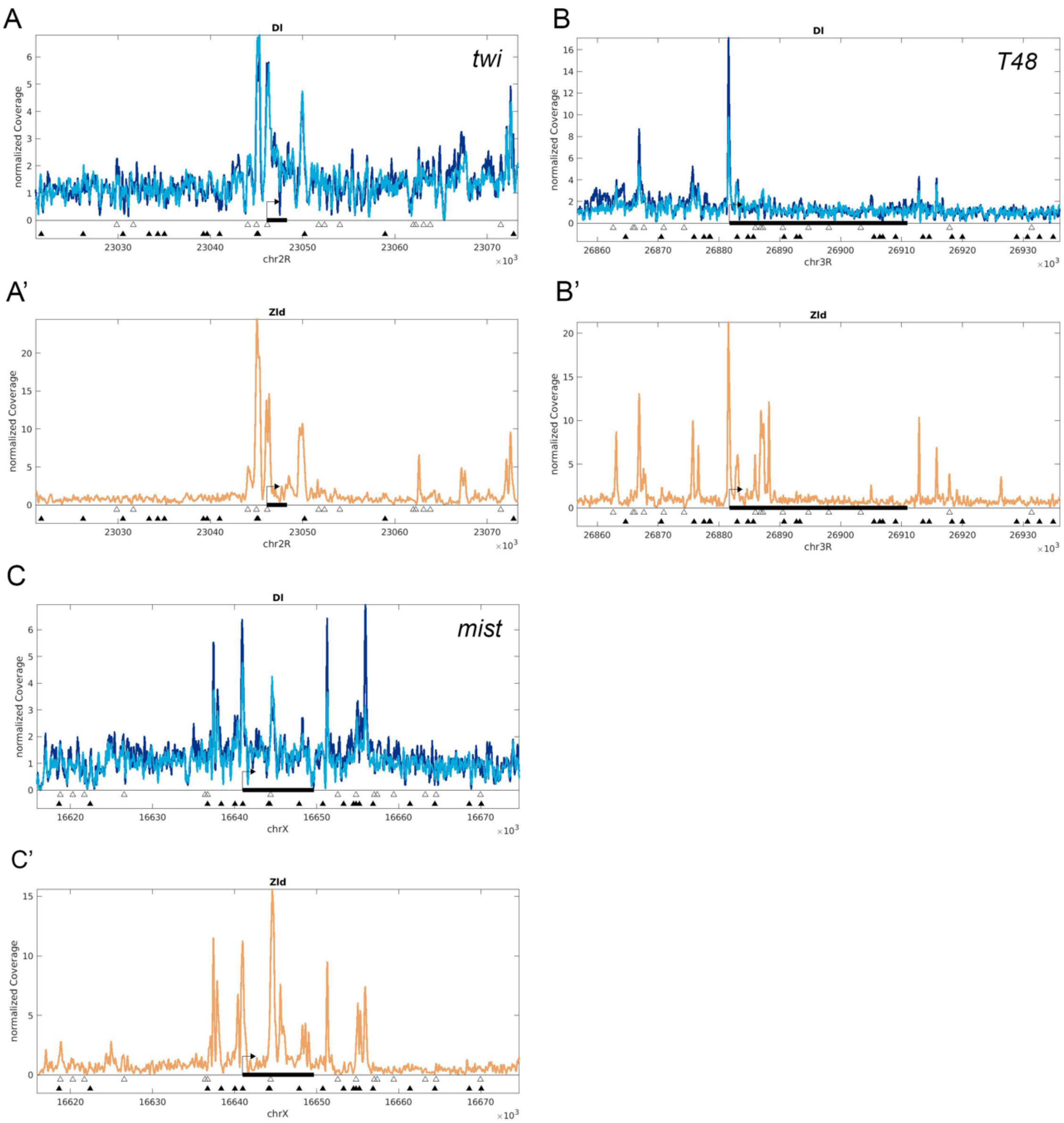

Fig. S2. Binding of Dorsal and Zelda

CHIP seq data from (Sun et al., 2015) was plotted. Right-pointing arrows mark transcription start sites and the extent of the transcribed regions are shown by bold black lines. A) Binding of Dorsal to the *twi* regulatory region. A') Binding of Zelda to the *twi* regulatory region. Prominent binding of both is detected immediately upstream to the TS. B,B') Binding of Dorsal and Zelda to the *T48* regulatory the region. Prominent binding is detected upstream to the TS and in the first intron. C.C') Binding of Dorsal and Zelda to the *mist* regulatory region. Prominent binding is detected upstream to the TS and for Zld also within the transcribed region.

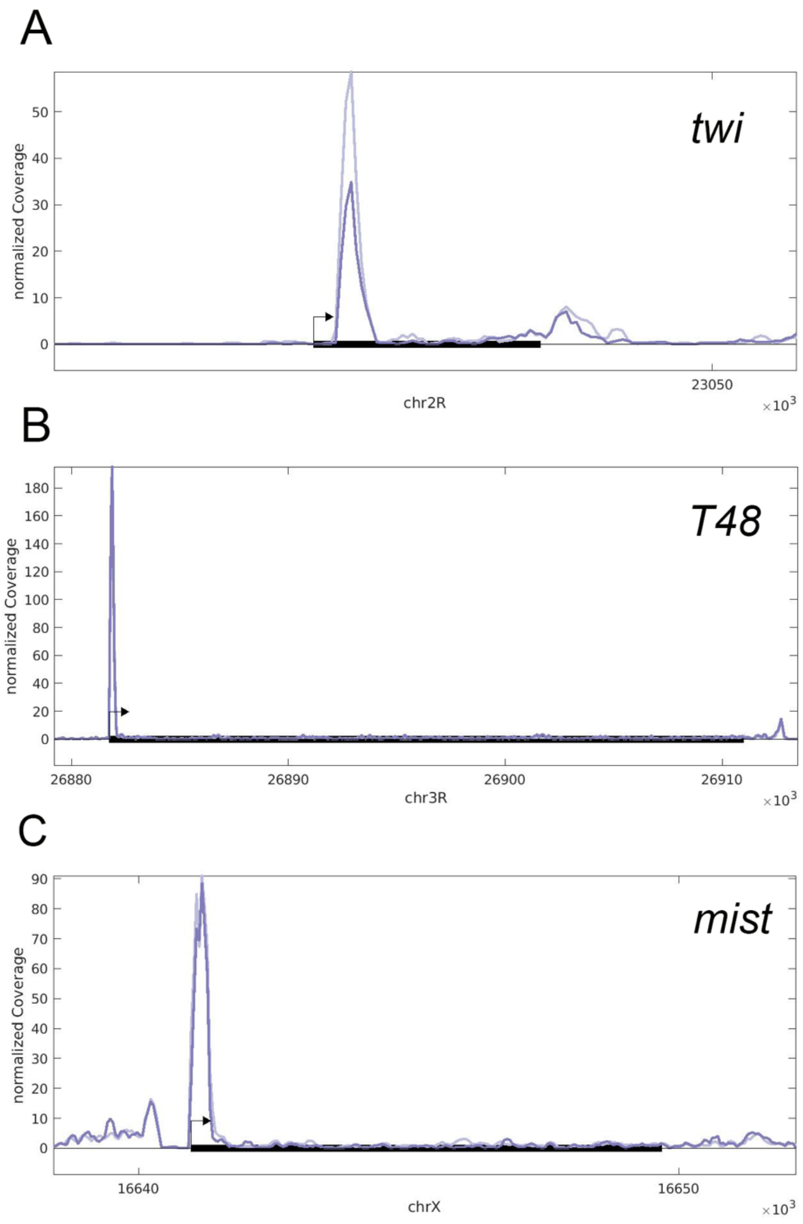

Fig. S3. Pol II Pausing

Chip seq data of Pol II from (Saunders et al., 2013) was plotted. Right-pointing arrows mark transcription start sites and the extent of the transcribed regions are shown by bold black lines. Prominent stalling was observed. A) *twi.* B) *T48.* C) *mist.*

Development • Supplementary information

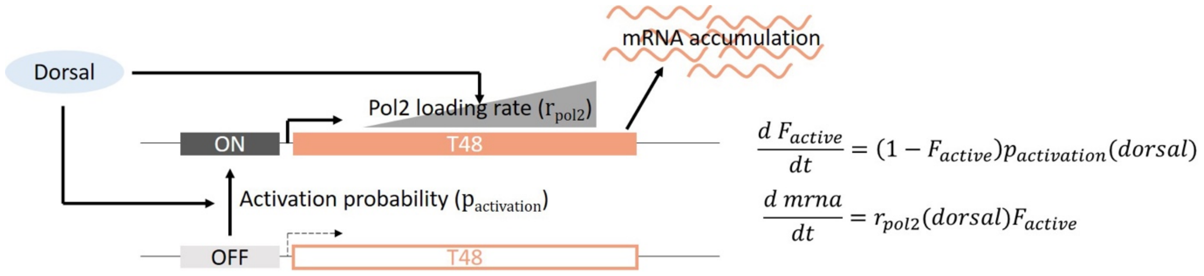

Fig. S4. Modelling Dorsal-dependent mRNA accumulation

Based on experimental results, Dorsal was assumed to determine both the promoter activation probability and Pol II loading rate of the *T48* gene. Mature transcripts are assumed to be stable and accumulate during the course of the simulation (~15 minutes).

Differential equations used to simulate the dynamical system- Activation probability ($p_{activation}$) and Pol II loading rate (r_{pol2}) depend linearly on Dorsal level and thus on the position along the DV axis. The dynamics of the activated promoter fraction ($F_{activated}$) and the mRNA level (mrna) is simulated for 15 minutes after model initiation (no mRNA or activated promoters at time 0). Model parameters were either arbitrary (r_{pol2} at maximal Dorsal levels) or based on experimental results ($p_{activation}$ at maximal Dorsal=0.07 min^{-1}) (Ambrosi et al., 2014).

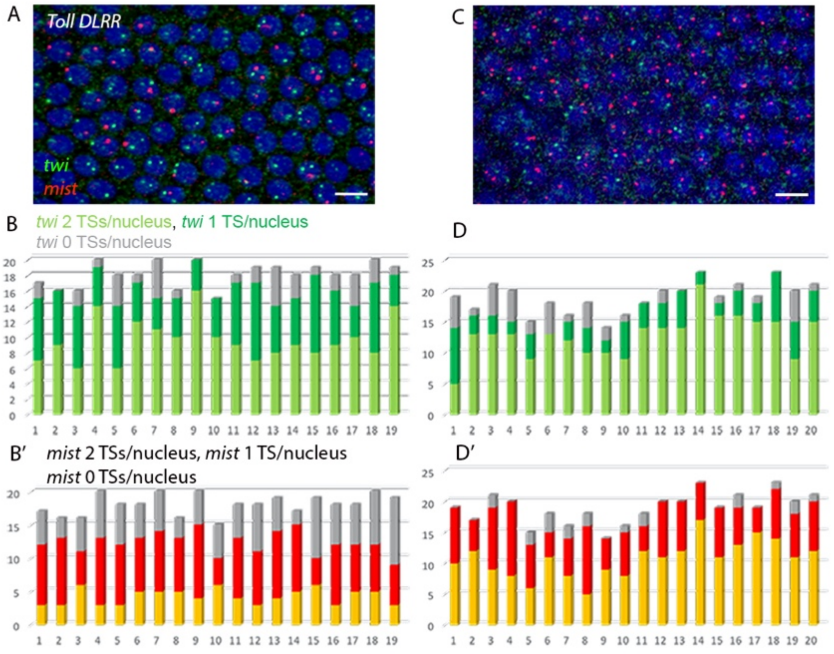

Fig. S5. Uniform activation of *mist* in *Toll$^{\Delta LRR}$* embryos

Uniform expression of the constitutive Toll$^{\Delta LRR}$ construct drives activation of the pathway and nuclear targeting of Dorsal along the entire embryo circumference. Induction of *twi* (green) and *mist* (red) TSs responds accordingly. In a younger embryo (A-B') one or two *twi* TSs are observed in most nuclei, while only a single or no *mist* TSs are observed in many nuclei. In an older embryo (C-D') the majority of nuclei exhibit two TSs for both genes. Scale bar 10 μm.

Development • Supplementary information

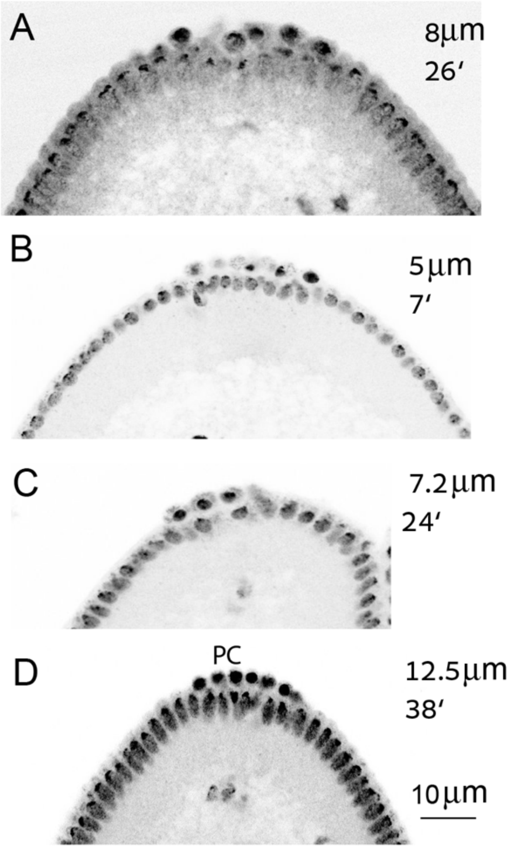

Fig. S6. Determination of embryo age at NC 14
The reported apical-basal lengths of DAPI-stained nuclei were calculated as the average length of 20 nuclei on both sides of the pole cells (PC) . This value was used to estimate the embryo age in NC 14 at 20^{0}c, according to (Lecuit and Wieschaus, 2000). The embryos shown correspond to the panels in Figure 7. A) 8µm, B) 5.4 µm, C) 7.2 µm, D) 12.5 µm. Scale bar 10 µm.

Table S1. smFISH probes. Position of probes is marked from the first transcribed nucleotide.

Gene	Probe	# Probes	Flurofour
twist	53-2,362	48	Quasar 670
T48	1-29,056	48	Quasar 570
T48 5'	1-2,491	48	Quasar 570
T48 intron	13,800-16,474	48	Cal-Fluor-Red 610
T48 3'	27,796-20,056	48	Quasar 670
mist 5'	6-2,524	48	Quasar 570

Development • Supplementary information

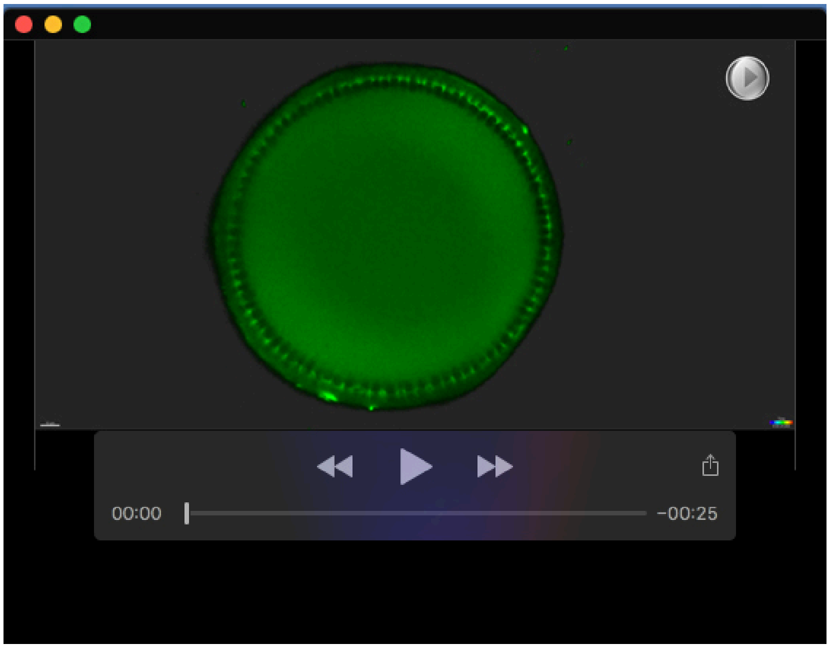

Movie 1. Dynamics of Myosin II recruitment
The dynamic localization pattern of Myosin II during early NC 14, monitored using a GFP "protein-trap" in the Myosin II heavy-chain (*zipper*) gene locus, as visualized in cross section by Lightsheet microscopy. During the process of cellularization, Myosin II is initially recruited to the basal aspect of the future cell membranes as they extend from the surface. Upon completion of cellularization, Myosin II disappears form the basal position and is recruited to the apical side in the ventral-most cells, where it drives furrow formation. The graded pattern of Myosin II recruitment within the ventral domain shapes the pattern of cell invagination. The movie was obtained at a rate of 1 frame/min.

Development • Supplementary information

References

Ambrosi, P., Chahda, J. S., Koslen, H. R., Chiel, H. J. and Mizutani, C. M. (2014). Modeling of the dorsal gradient across species reveals interaction between embryo morphology and Toll signaling pathway during evolution. *PLoS Comput Biol* **10**, e1003807.

Lecuit, T. and Wieschaus, E. (2000). Polarized insertion of new membrane from a cytoplasmic reservoir during cleavage of the Drosophila embryo. *J Cell Biol* **150**, 849-860.

Saunders, A., Core, L. J., Sutcliffe, C., Lis, J. T. and Ashe, H. L. (2013). Extensive polymerase pausing during Drosophila axis patterning enables high-level and pliable transcription. *Genes Dev* **27**, 1146-1158.

Sun, Y., Nien, C. Y., Chen, K., Liu, H. Y., Johnston, J., Zeitlinger, J. and Rushlow, C. (2015). Zelda overcomes the high intrinsic nucleosome barrier at enhancers during Drosophila zygotic genome activation. *Genome Res* **25**, 1703-1714.
