# Supplementary material for: Generation and timing of graded responses to morphogen gradients
Source: Development. 2021 Dec 17;148(24):dev199991. doi: 10.1242/dev.199991 (PMC8722393; doi:10.1242/dev.199991)
Supplement: Reviewer comments [file develop-148-199991_review_history.pdf]

Generation and timing of graded responses to morphogen gradients

Shari Carmon, Felix Jonas, Naama Barkai, Eyal D. Schejter and Ben-Zion Shilo
DOI: 10.1242/dev.199991

Editor: Thomas Lecuit

Review timeline
Original submission: 12 July 2021
Editorial decision: 13 September 2021
First revision received: 19 October 2021
Accepted: 17 November 2021

Original submission

First decision letter

MS ID#: DEVELOP/2021/199991

MS TITLE: Generation and timing of graded responses to morphogen gradients

AUTHORS: Shari Carmon, Felix Jonas, Naama Barkai, Eyal D Schejter, and Ben-Zion Shilo

I apologise for the delay beforre coming back to you. I have now received all the referees reports on the above manuscript, and have reached a decision. The referees' comments are appended below, or you can access them online: please go to BenchPress and click on the 'Manuscripts with Decisions' queue in the Author Area.

The overall evaluation is positive and we would like to publish a revised manuscript in Development, provided that the referees' comments can be satisfactorily addressed. There are a number of suggestions to improve clarity of the paper and also some suggestions from Referee 1 for additional data. The one that I find the most important is obtaining some functional evidence that the length of the intron is important for the regulation of T48 expression pattern, which is also mentioned by Ref 2. I appreciate that it may be quite difficult to do though. Please let me know what you can do. The alternative would be to discuss the limits of the study given this caveat.

We are aware that you may currently be unable to access the lab to undertake experimental revisions. If it would be helpful, we encourage you to contact us to discuss your revision in greater detail. Please send us a point-by-point response indicating where you are able to address concerns raised (either experimentally or by changes to the text) and where you will not be able to do so within the normal timeframe of a revision. We will then provide further guidance. Please also note that we are happy to extend revision timeframes as necessary.

Reviewer 1

Advance summary and potential significance to field

The manuscript by Carmon et al investigates the mechanisms underlying the graded response to a morphogen gradient. Specifically, they monitor the transcriptional response of two Dorsal targets, T48 and mist during mesoderm formation in Drosophila early embryos. For this, they employ single molecule FISH (smFISH) on wild type and ventralized embryos at various timing of mesoderm induction.

© 2021. Published by The Company of Biologists under the terms of the Creative Commons Attribution License (https://creativecommons.org/licenses/by/4.0/).

The major finding of this manuscript is that the gradual response of T48/Mist relies on two complementary mechanisms:
1) the priming of these genes by Dorsal
2) the loading rate of Pol II, also dependent on Dorsal nuclear levels.

The data are of high quality and represent solid quantitative information, of interest to a broad audience of developmental biologists who study mechanisms of gene expression regulation.

Comments for the author

Major comments:
1) A weakness that I find is the lack of functional analysis to validate some of the conclusions made from the quantitative analysis of the transcriptional response.
For example, it would be exciting to assess the functional consequences of an intronless T48 gene by CRISPR gene editing. Phenotypes in terms of timing/coordination in the constriction of the actomyosin network would greatly improve the biological significance of the transcriptional findings of this manuscript.
2) The effect of intron size on T48 expression is highly dependent on Pol II elongation speed. As the authors have not directly measured this spped, the conclusions should be nuanced. Indeed the 2kb/min elongation speed considered by the authors (Fukaya et al 2017), has been obtained on a reporter gene, not on the endogenous T48 locus. Moreover, Pol II elongation speed is known to vary from gene to gene. In principle, Pol II velocity could be higher than 2kb/min for extremely long genes. This point should be discussed. Maybe simulations with other elongation speed could help.
3) A major finding from this work is that timing is a central parameter responsible for coordinating cellular movement during gastrulation. However, the manuscript only examines the timing of mRNA production and does not assess consequences in terms of protein dynamics. Ideally genetic tools like Llama-Tag or fast folding GFP would help elucidate T48/Fog or Mist protein dynamics, but classical antibody staining in very precisely staged embryos (with membrane invagination) could be useful too. This would significantly increase the scope of the manuscript.

Minor points:
- Since T48-MS2 genetic tools exist, it would have been good to confirm the estimated Pol II loading rates from smFISH by Live-imaging data.

-The introduction provides lots of details regarding the cellular aspects of gastrulation and on the establishment of dorsal gradient. Since the manuscript is centered on quantification of a transcriptional response, I feel that potential underlying mechanisms should be better introduced.
-To my point of view, Figure 1 should be a supplementary figure. Indeed, as the manuscript does not test functional aspects of the main players driving gastrulation, it seems inappropriate to emphasize these players as a main figure. I would replace this figure by a clear schematic of the gene regulatory network assessed in this study, with important information regarding the regulation of Twi, T48 and mist by Dorsal, Twi and Zelda transcription factors.
-The sentence regarding the appearance of Twi protein should be modified: yes Twi protein seems to appear uniformly but it does not appear ‘early’. In fact Twi nuclear protein is not detected until mid nc14 (Dufourt et al., 2021).

Reviewer 2

Advance summary and potential significance to field

This manuscript from Carmon et al. reports on the transcriptional initiation and transcript residence of DV target genes in the fly embryo. The authors examine the transcriptional profile of Twist, which displays a more bistable switched state, and the profiles of the graded and zygotically expressed T48 and Mist genes. The Twist characterization serves as a nice control for the T48 and Mist data. The authors suggest that graded Dorsal may produce the graded profiles of T48 and Mist, although this is not conclusively shown. However, the analysis of the temporal dynamics of Twist, T48, and Mist provide excellent insights into how transcriptional initiation of these early-expressed genes occurs, and the analysis in Tl mutant embryos is also suggestive of a possibly direct regulation. The authors then go on to examine if long introns found in T48 and Mist delay the end of

© 2021. Published by The Company of Biologists under the terms of the Creative Commons Attribution License (https://creativecommons.org/licenses/by/4.0/).

transcript production and, potentially, the activation of apical actomyosin contractility. Again, although the data on transcript production times is convincing, the final hypothesis (that long introns contribute to a delay in actomyosin activation) is not conclusively shown.
However, this data is intriguing as well. The analysis is conducted in fixed and stained embryos, but is performed well, and the data is convincing. The writing is also nicely done, with a few exceptions. In general, this seems to be a nice study that would make a good addition to Development. A few more detailed comments follow:

Comments for the author

1) Is there data for how often two loci are observed for a general house-keeping gene with the authors' methodology? I wasn't sure if the Twist data would be substantially different once it was at steady state from a general, highly expressed gene.
2) The long intron data felt a little tacked on – it is interesting, but it is difficult for the authors to truly demonstrate their hypothesis that long introns are responsible for the delay in apical actomyosin function. This should be discussed further in the Discussion/Results. Also, I suspect this is not the case or it would have probably been shown, but does the T48 cDNA construct drive earlier apical actomyosin and/contractility? Perhaps the Mist cDNA would also need to be present? If these experiments worked, it would seem to potentially be a separate manuscript, and this data could be removed. Entirely up to the authors, of course, but greater clarity on these issues should be provided.
3) page 8, the authors use "younger" and "older" to describe the timing of the embryos – I found this too inexact and it would be better to use clearer definitions.
4) 4E – is the P=0.47 a typo? Seems difficult to claim dependence if not.

Minor notes:
a) "naïve" is spelled differently in two places on page 1.
b) typo, "encloding" page 4
c) Awkward language page 4, "which allows to visualize both the active transcription sites"

<u>Reviewer 3</u>

Advance summary and potential significance to field

This is a very clear quantitative study of dynamic responses to the Dorsal morphogen gradient, focusing on the distinctions between all or none response displayed by twist and graded responses of T48, mist, and myosin localization. The presented measurements of transcriptional responses are quite convincing. When it comes to myosin localization, I am surprised that the authors do not consider the fact an additional layer of regulation supplied by secreted Fog, which can easily contribute to the graded response. I would also suggest adding a simple kinetic model to the Discussion section. All in all, the study is well suited for Development.

Comments for the author

This is a very clear quantitative study of dynamic responses to the Dorsal morphogen gradient, focusing on the distinctions between all or none response displayed by twist and graded responses of T48, mist, and myosin localization. The presented measurements of transcriptional responses are quite convincing. When it comes to myosin localization, I am surprised that the authors do not consider the fact an additional layer of regulation supplied by secreted Fog, which can easily contribute to the graded response. I would also suggest adding a simple kinetic model to the Discussion section. All in all, the study is well suited for Development.

© 2021. Published by The Company of Biologists under the terms of the Creative Commons Attribution License (https://creativecommons.org/licenses/by/4.0/).

First revision

Author response to reviewers' comments

We thank the reviewers for their constructive comments, and are glad that you liked our manuscript.
Below please find a point-by-point response to the comments, outlining the changes we have made.

Reviewer 1 Comments for the author

Major comments:
1) A weakness that I find is the lack of functional analysis to validate some of the conclusions made from the quantitative analysis of the transcriptional response.
For example, it would be exciting to assess the functional consequences of an intronless T48 gene by CRISPR gene editing. Phenotypes in terms of timing/coordination in the constriction of the actomyosin network would greatly improve the biological significance of the transcriptional findings of this manuscript.

We have gone to great lengths to test the effect of T48 intron size on the timing of gastrulation. A CRISPR excision of the large intron was designed, and around 300 resulting lines were screened for intron excision. All of them retained the original intron. At this point, we could not determine if the result indicated a lethal effect of the excised intron or inefficient activity of the guide RNAs we used. We constructed a conditional allele based on the T48 enhancer and the T48 cDNA, with an FRT stop cassette. Following excision of the stop cassette, the flies were viable and are the ones we used to follow the kinetics of intronless T48 transcription in Figure 7. The lack of functional outcome could be attributed to a lower expression level compared to the endogenous locus, and/or to the reliance on additional factors for timely invagination of the mesoderm. In support of this notion, the phenotypes of T48 or fog mutants alone are mild (e.g. Kolsch et al., 2007; Leptin, 1991). To follow the direct consequence of early T48 transcription termination and translation, we attempted to monitor the apical recruitment of RhoGEF2-GFP. However, the signal of this reporter was too weak in our hands, and the lower expression level of the T48 cDNA transgene may again compromise the recruitment and detection capacity.

2) The effect of intron size on T48 expression is highly dependent on Pol II elongation speed. As the authors have not directly measured this spped, the conclusions should be nuanced. Indeed the 2kb/min elongation speed considered by the authors (Fukaya et al 2017), has been obtained on a reporter gene, not on the endogenous T48 locus. Moreover, Pol II elongation speed is known to vary from gene to gene. In principle, Pol II velocity could be higher than 2kb/min for extremely long genes. This point should be discussed. Maybe simulations with other elongation speed could help.

Pol II elongation speed can be indirectly deduced from an earlier work by Lim et al. (CB 2016), that detected a 30-minute difference between the signal of a 5' labeled T48 and a 3' labeled fog. In order to provide a more direct measurement of transcription duration, we have repeated the experiment in Figure 7, taking precise measurements of the apical-basal length of the nuclei as a proxy for age within NC 14 at 20 degrees C (according to Lecuit et al., 2000). The updated data shows that the time difference between the appearance of T48 TSs displaying the 5' and 3' probes is ~17 minutes. We also find it interesting that the introns of T48 and fog are similar in size, and that this size is conserved among the different Drosophila species.
The Pol II speed would determine the timing of translation onset, but not the underlying distribution of mRNA. Our simulations, therefore, focused on the generation of graded mRNA patterns, and did not incorporate the duration of transcription for each Pol II complex.

3) A major finding from this work is that timing is a central parameter responsible for coordinating cellular movement during gastrulation. However, the manuscript only examines the timing of mRNA production and does not assess consequences in terms of protein dynamics. Ideally genetic tools like Llama-Tag or fast folding GFP would help elucidate T48/Fog or Mist protein dynamics, but classical antibody staining in very precisely staged embryos (with membrane invagination) could be useful too. This would significantly increase the scope of the manuscript.

© 2021. Published by The Company of Biologists under the terms of the Creative Commons Attribution License (https://creativecommons.org/licenses/by/4.0/).

While measuring the corresponding protein levels would provide an additional facet to the work, we believe it is beyond the scope of this paper which focuses on transcriptional mechanisms for generating graded responses.

Minor points:

- Since T48-MS2 genetic tools exist, it would have been good to confirm the estimated Pol II loading rates from smFISH by Live-imaging data.

The T48-MS2 construct generated by Lim et al. (2017) harbors the yellow gene downstream of the promoter. We, therefore, preferred to carry out the analysis on the endogenous T48 and mist loci, albeit in fixed embryos.

-The introduction provides lots of details regarding the cellular aspects of gastrulation and on the establishment of dorsal gradient. Since the manuscript is centered on quantification of a transcriptional response, I feel that potential underlying mechanisms should be better introduced.

-To my point of view, Figure 1 should be a supplementary figure. Indeed, as the manuscript does not test functional aspects of the main players driving gastrulation, it seems inappropriate to emphasize these players as a main figure. I would replace this figure by a clear schematic of the gene regulatory network assessed in this study, with important information regarding the regulation of Twi, T48 and mist by Dorsal, Twi and Zelda transcription factors.

We have split Figure 1, such that the scheme of apical constriction is now a Supplementary figure. However, we feel it is important to highlight which elements in the Myosin II cascade are maternal and which ones are zygotic (now Figure 1A). We have added a panel to the figure (B) outlining the regulatory transcriptional network we address.

-The sentence regarding the appearance of Twi protein should be modified: yes Twi protein seems to appear uniformly but it does not appear ‘early’. In fact Twi nuclear protein is not detected until mid nc14 (Dufourt et al., 2021).

The Dufout paper (Fig. 2D) shows two steps of Twist protein accumulation in NC 14. The first phase plateaus by ~ 5 minutes (which would qualify as early), and the next rise is only at ~30-40 minutes, which is beyond the relevant time for the transcriptional responses we study.

Reviewer 2 Comments for the author
1) Is there data for how often two loci are observed for a general house-keeping gene with the authors' methodology? I wasn't sure if the Twist data would be substantially different once it was at steady state from a general, highly expressed gene.

The burst size of housekeeping genes may depend on the stability of the products to maintain a steady-state. Perhaps a better control is the result we obtained following ubiquitous activation of the Toll pathway, demonstrating that under these conditions the three genes we tested eventually activate both loci (Figures 3, S5).

2) The long intron data felt a little tacked on – it is interesting, but it is difficult for the authors to truly demonstrate their hypothesis that long introns are responsible for the delay in apical actomyosin function. This should be discussed further in the Discussion/Results. Also, I suspect this is not the case or it would have probably been shown, but does the T48 cDNA construct drive earlier apical actomyosin and/contractility? Perhaps the Mist cDNA would also need to be present? If these experiments worked, it would seem to potentially be a separate manuscript, and this data could be removed. Entirely up to the authors, of course, but greater clarity on these issues should be provided.

See our detailed response to Reviewer 1 on the same issue.

3) page 8, the authors use “younger” and “older” to describe the timing of the embryos – I found this too inexact and it would be better to use clearer definitions.

© 2021. Published by The Company of Biologists under the terms of the Creative Commons Attribution License (https://creativecommons.org/licenses/by/4.0/).

We have now added the estimated age of the embryos based on nuclear morphology (Lecuit et al., 2000), and used this to provide an estimate for the duration of T48 transcription in Figure 7 B-D.

4) 4E – is the P=0.47 a typo? Seems difficult to claim dependence if not.

This is a Pearson correlation reflecting a high correlation level.

Minor notes:

a) “naïve” is spelled differently in two places on page 1.

Corrected

b) typo, “encloding” page 4

Corrected

c) Awkward language page 4, “which allows to visualize both the active transcription sites“

Corrected

Reviewer 3 Comments for the author
This is a very clear quantitative study of dynamic responses to the Dorsal morphogen gradient, focusing on the distinctions between all or none response displayed by twist and graded responses of T48, mist, and myosin localization. The presented measurements of transcriptional responses are quite convincing. When it comes to myosin localization, I am surprised that the authors do not consider the fact an additional layer of regulation supplied by secreted Fog, which can easily contribute to the graded response. I would also suggest adding a simple kinetic model to the Discussion section. All in all, the study is well suited for Development.

We have looked at fog transcription in detail by smFISH, and find it less stringently regulated than T48 or mist. Initially fog expression is very sporadic and covers nuclei in the lateral mesoderm as well. Subsequently, it displays the ventral to lateral expansion, but still in a “salt and pepper” fashion. Keeping in mind that the Fog protein is secreted, in contrast to T48 and Mist which are membrane proteins that function in a cell-autonomous fashion, it may not be necessary for all nuclei to express the fog gene, since the subsequent secretion to the fluid extracellular space may equilibrate the distribution of the Fog protein. However, this extracellular dispersion should disrupt any graded distribution of Fog protein. We thus believe that Fog may act as a temporal switch for Mist activation, but the pattern of activation would be driven primarily by the distribution of Mist. We briefly relate to these issues in the text.

Second decision letter

MS ID#: DEVELOP/2021/199991

MS TITLE: Generation and timing of graded responses to morphogen gradients

AUTHORS: Shari Carmon, Felix Jonas, Naama Barkai, Eyal D Schejter, and Ben-Zion Shilo
ARTICLE TYPE: Research Article

I am happy to tell you that your manuscript has been accepted for publication in Development, pending our standard ethics checks. During the proof, please pay attention to write R=3D0.47 instead of P=3D0.47 in legend of Figure 4E, since, as pointed out by Rev 2 under their point 2, this is a typo, and you meant Pearson coefficient R.

© 2021. Published by The Company of Biologists under the terms of the Creative Commons Attribution License (https://creativecommons.org/licenses/by/4.0/).

<u>Reviewer 1</u>

Advance summary and potential significance to field

The revised manuscript and the letter to the reviewers addressed all the concerns I had raised in the initial submission. I recommend publication of the manuscript. Congratulations for this nice piece of work !

Comments for the author

N/A

<u>Reviewer 2</u>

Advance summary and potential significance to field

I found the author's response to reviewer's comments to be unsatisfactory. Again, as in the first review of the paper, I think the analysis of the temporal dynamics of Twist, T48, and Mist provide excellent insights into how transcriptional initiation of these early-expressed genes occurs. The data on long introns potentially delaying actomyosin contractility is also intriguing, although not conclusive. As discussed below requested reviewer clarifications were not performed, even in cases where two reviewers had similar concerns. More details are below.

Comments for the author

As mentioned above, I found the author's response to reviewer's comment to be inadequate. Although this is a potentially nice study, it seemed the authors made little effort to address the reviewer comments. In some cases, this could have been as simple as straightforward textual edits.

1) As two different reviewers pointed out, one of the main final hypotheses (that long introns contribute to a delay in actomyosin activation) is not conclusively shown and relies on correlative behaviors. This could be explicitly tested by looking at protein activity/localization and an available transgene, and does not seem like a difficult thing to do, but the authors simply reply with a "this is beyond the scope of the study".
Indeed, the author's own manuscript says, "In addition to the expression pattern of these two regulators the timing of their ACTIVITY [caps added] is also critical, since the cell movements of gastrulation should commence only after the completion of cellularization. We find that such timing is dictated by long introns found in the T48 and fog genes, which delay the appearance of mature mRNAs that can undergo translation." Even if the author's do not wish to do these experiments, they do need to take the time to qualify their statements and point out these fundamental weaknesses in their results and discussion. I have re-pasted my comments from the first review at the end (which the authors' did not directly reply to in their rebuttal letter).

2) Also, very simple text edits could have made. As this reviewer pointed out, it is easy to see, "p=0.47" in 4E and think that this is a typo for significance measurement. The authors' have simply replied with "this is a pearson's correlation coefficient", but this is never stated in the figure legend, nor have the authors done a simple edit to clarify this. Typically, these values are reported with an "r=x value" to avoid just such confusion.
<re-pasted comments from first review>

3) The long intron data felt a little tacked on – it is interesting, but it is difficult for the authors to truly demonstrate their hypothesis that long introns are responsible for the delay in apical actomyosin function.
This should be discussed further in the Discussion/Results. Also, I suspect this is not the case or it would have probably been shown, but does the T48 cDNA construct drive earlier apical actomyosin and/contractility? Perhaps the Mist cDNA would also need to be present? <added -- or these future potential experiments could be briefly discussed in the Discussion> If these experiments worked, it would seem to potentially be a separate manuscript, and this data could be removed. Entirely up to the authors, of course, but greater clarity on these issues should be provided.

© 2021. Published by The Company of Biologists under the terms of the Creative Commons Attribution License (https://creativecommons.org/licenses/by/4.0/).
